# Why we should raise stroke awareness in the younger population?

**DOI:** 10.1111/cns.14067

**Published:** 2023-01-11

**Authors:** Jing Zhao, Jing Yuan, Kevin Lu, Anthony Rudd, Renyu Liu

**Affiliations:** ^1^ Department of Neurology, Minhang Hospital Fudan University Shanghai China; ^2^ Minhang Hospital, School of Pharmacy Fudan University Shanghai China; ^3^ University of South Carolina Columbia South Carolina USA; ^4^ Stroke Medicine Kings College London London UK; ^5^ Departments of Anesthesiology and Critical Care, and Neurology Perelman School of Medicine at the University of Pennsylvania Philadelphia Pennsylvania USA

Julie Chin, a news anchor in Oklahoma, had a possible stroke during her live TV broadcast. This episode triggered a massive wave of news reports across the world. Many major news networks (including The New York Times and The Guardian), and many social media outlets reported the incidence. A search in Google using the keywords of “news anchor Oklahoma Julie Chin stroke” returned over 1,010,000 results (accessed on September 16th, 2022). This story triggered such widespread attention not just because she is a news anchor, but because she is very young, only in her 40s, and her colleagues acted immediately by calling 911. Stroke remains the second leading cause of death and disability worldwide. A stroke occurs every 40 s in the United States, and every 12 s in China. It is rare that a stroke story causes such media attention unless the story relates to a celebrity or is very unique. In the general public's view, stroke occurs only in the elderly. Unfortunately, stroke in young age adults is not rare. Stroke in the young is a trending global crisis since it is estimated that 3.6 million young people suffer from ischemic stroke every year. The average age of first stroke is getting younger especially in developing countries.^1^, ^2^ While supportive therapy (e.g., blood pressure management) and time‐sensitive thrombolysis and thrombectomy are available for ischemic stroke, the use of such potentially life‐saving therapies remains very low, at <10%, of stroke victims even in well‐developed health systems. This is partly due to the long prehospital delay, especially in non‐English‐speaking populations.

In Julie's case, it is likely that a major catastrophe was prevented due to the quick response by her colleagues. Many other young stroke patients are not as lucky. Because it is not well‐recognized that stroke can occur in young adults, it is often not identified quickly and managed properly by calling for an ambulance. Therefore, time‐sensitive life‐saving therapies may not be able to be delivered because of unfortunate and needless delays. In Julie's case, speech disturbance was a major sign. Slurred speech occurs in >70% of stroke patients.^3^

Dr. Lei Wang, a postdoc student in West Virginia, contacted us early this year and authorized us to share his stroke story for educational purposes. On February 2, 2022 at around 4 a.m., he woke up to find his right leg was numb, but did not realize that he was having a possible stroke. He went back to sleep until 8 o'clock. After getting out of bed, his right leg was weak, he could not brush his teeth with his right hand, and he could not speak clearly. He then realized that he might be having a stroke. He called his roommate and decided to drive to the urgent care clinic nearby. After arriving at the clinic, the medical staff recommended that he takes himself to the nearby hospital immediately. An ischemic stroke was later confirmed. However, he had missed the time window for intravenous thrombolysis therapy and was not eligible for thrombectomy. He was discharged to rehabilitation and started a lengthy recovery for his neurological disability. Fortunately, his recovery went well and he volunteered to share his story at our stroke educational event in May 2022 in Philadelphia. During the event, he spoke emotionally to emphasize that people should avoid his mistakes, such as not calling for an ambulance and not controlling his blood pressure (Figure 1A).

**FIGURE 1:**
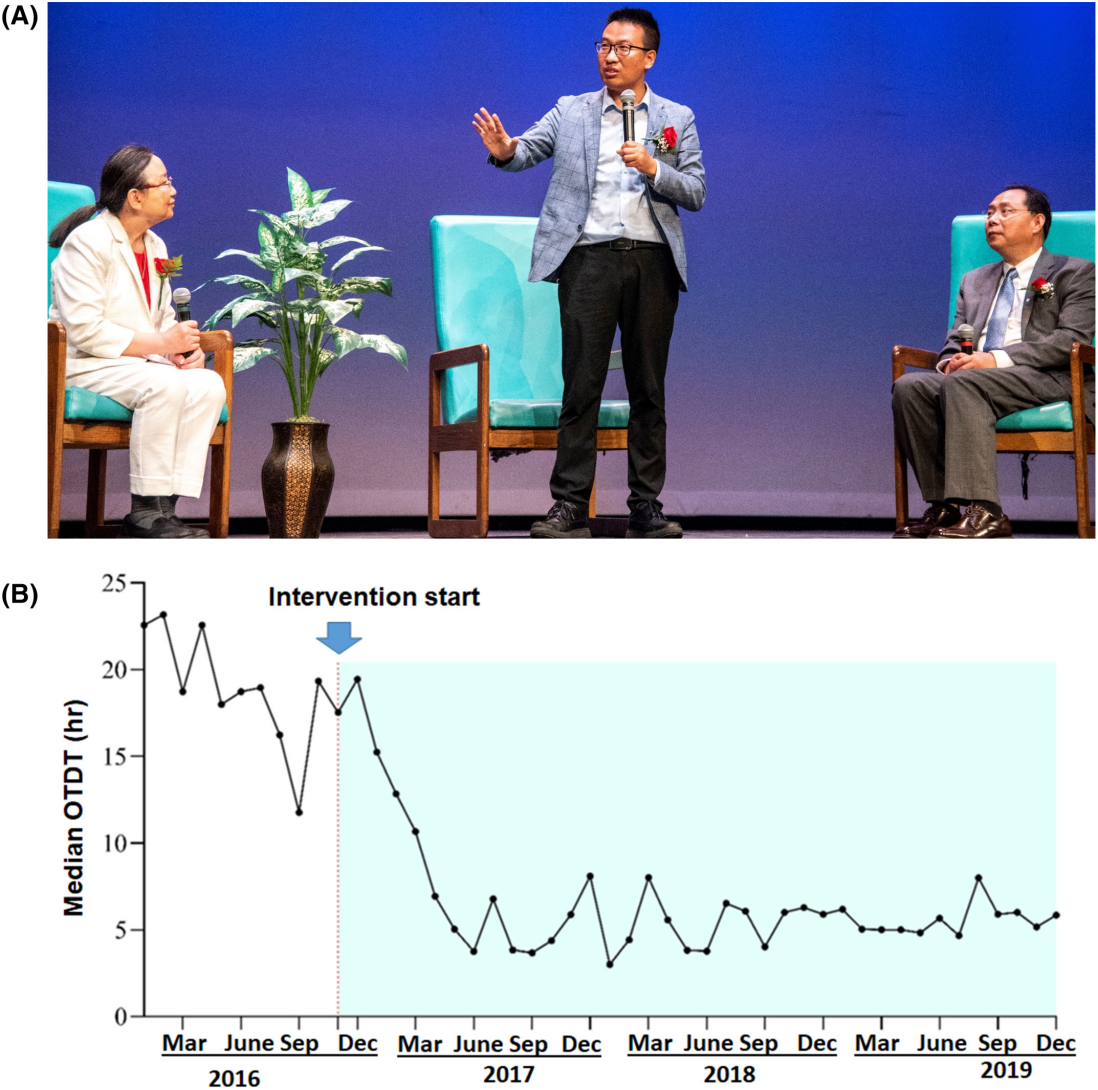
(A) Dr. Lei Wang, (standing) a young stroke survivor shares his stroke story with the audience at the Stoke 911 educational event held in Philadelphia in May 23, 2022, Left, Dr. Xiaobin Li; right, Dr. Renyu Liu. (B) Example of success in reduction of stroke prehospital delay in a community in Shanghai, China from 2016 to 2019, using a culture‐adapted multifaceted stroke awareness campaign (Stroke 120), including a family‐based educational program targeting young adults. The median OTDT decreased from 18.72 (7.44–27.84) h to 6.00 (2.00–16.35) h. Arrow point is the starting point of the educational intervention. OTDT, the onset‐to‐door time, or the prehospital delay time.

While there are many anecdotal news reports about stroke in the young, stroke occurs more frequently with increasing age. Therefore, current stroke education is more focused on the elderly population (65 and older). With the increase of stroke in the young and the fact that younger patients are less likely to call emergency services, future educational efforts should have a significant shift toward the younger population (<65 years old), which may result in a larger, and potentially more long‐lasting impact. The initial focus could potentially be on those who have family history or risk factors for stroke such as hypertension and diabetes. The current impression is that younger stroke patients have longer prehospital delay than elderly patients. This finding needs to be confirmed with large datasets and the mechanism of possible age disparity needs to be further studied.

Stroke awareness remains suboptimal across the world, even in well‐developed countries, and especially in young people. Based on a recent study, 2.7% of young adults, representing 2.9 million people in the United States, did not know any stroke symptoms.^4^ Clear guidance exists for the structure of hospital stroke services but there is much less guidance for prehospital care. A well‐coordinated effort is needed globally to improve the effectiveness of prehospital care and to provide a consistent message understandable to the majority of societies and cultures. Country‐ or region‐specific strategies are also needed, by incorporating consideration of both language and culture barriers.^5^, ^6^, ^7^ To overcome deficiencies in stroke prehospital care, specific taskforces might be an effective strategy, especially in developing countries.^8^ We worked with the World Stroke Organization to establish The World Stroke Organization Taskforce for Prehospital Care (WSOTPC). The mission of this group is to reduce the global burden of stroke with a special focus on prehospital stroke care. The goal is to improve stroke prehospital care, which includes stroke awareness, optimizing processes, developing novel tools, advocating policy, and optimizing transition from prehospital care to in‐hospital care. We have been shifting our educational efforts toward young people and have hosted multiple meetings where we invite young stroke survivors to speak at our educational events. We have been producing short movies based on true stories of stroke in the young. We strongly believe that there needs to be an emphasis on stroke awareness in younger populations (in addition to continuing to educate the older population) and to use language and culture‐adapted strategies to improve stroke awareness.^9^ Our recent study clearly indicated that a culture‐adapted multifaceted stroke awareness campaign (Stroke 120), including a family‐based educational program targeting young adults, could decrease stroke prehospital delay significantly, the median onset to door time decreased from 18.72 (7.44–27.84) h to 6.00 (2.00–16.35) h in a large community in Shanghai, China (Figure 1B).^10^

## FUNDING INFORMATION

National Natural Science Foundation of China; CIHR, Grant/Award Number: 81973157, PI: JZ. Funding from the University of Pennsylvania; Grant/Award Number: CREF‐030, PI: RL.

## CONFLICT OF INTEREST

All authors have no conflict of interest to declare.

## Data Availability

Data are available to a reasonable requestion by sending email to Dr. Renyu Liu at RenYu.Liu@pennmedicine.upenn.edu
